# Polymerizable Ionic Liquids for Solid-State Polymer Electrolytes

**DOI:** 10.3390/molecules24020324

**Published:** 2019-01-17

**Authors:** Robert Löwe, Thomas Hanemann, Andreas Hofmann

**Affiliations:** 1 Institute for Applied Materials, Karlsruhe Institute of Technology, Hermann-von-Helmholtz-Platz 1, D-76344 Eggenstein-Leopoldshafen, Germany; robert.loewe@kit.edu (R.L.); thomas.hanemann@kit.edu (T.H.); 2 Department of Microsystems Engineering, University of Freiburg, Georges-Köhler-Allee 102, D-79110 Freiburg, Germany

**Keywords:** polymerizable ionic liquids, property-structure relationship, conductivity, viscosity, density

## Abstract

Eight new polymerizable ammonium-TFSI ionic liquids were synthesized and characterized with respect to an application in energy storage devices. The ionic liquids feature methacrylate or acrylate termination as polymerizable groups. The preparation was optimized to obtain the precursors and ionic liquids in high yield. All products were characterized by NMR and IR spectroscopy. Phase transition temperatures were obtained by DSC analysis. Density, viscosity and ionic conductivity of the ionic liquids were compared and discussed. The results reveal that the length of attached alkyl groups as well as the methyl group at the polymerizable function have significant influences on the ionic liquids physicochemical properties. Ionic conductivity values vary between 0.264 mS cm^−1^ for [C_2_N_A,22_]TFSI and 0.080 mS cm^−1^ for [C_8_N_MA,22_]TFSI at 25 °C. Viscosity values are within a range of 0.762 Pa s for [C_2_N_A,22_]TFSI and 1.522 Pa s for [C_6_N_MA,22_]TFSI at 25 °C.

## 1. Introduction

Ionic liquids (ILs) possess a wide range of applications, being employed in CO_2_ adsorption [1], catalysis [2] or as solvents [3]. They are promising candidates in drug design [4] and drug delivery systems [5] due to their high biological activity [6] and the possibility to combine bioactive cations with bioactive anions, e.g., the salicylate (Sal) ionic liquid (IL) [EMIM-OSal][Sal] [7]. Within the field of energy storage systems, such as lithium ion batteries, ionic liquids are discussed as alternatives to conventional electrolytes [8]. Remarkable properties for electrochemical applications are low flammability, low vapor pressure, a broad electrochemical window (ca. 4–6 V) [8] and a high tunability of properties by structural variations and usage of IL mixtures.

For particular applications, immobilization of the ionic liquids is beneficial. In terms of drug design, a solid dosage form can lead to a higher convenience of products. In lithium ion batteries the electrolyte can move freely within the cell and thus lead to unwanted side reactions. A common example is the reactions of the aluminum current collector with bis(trifluoromethanesulfonyl)imide (TFSI) containing ionic liquids [9]. This type of degradation mechanism can be avoided if the electrolyte is fixed between the electrodes. Different approaches to immobilize ionic liquids are known. It is relatively easy to distinguish between materials in which the ILs are absorbed within a matrix [10] and materials in which the ionic liquids are fixed by covalent bonding [11,12]. The latter is an interesting approach for transferring the outstanding properties of ionic liquids to solid materials and covalent networks. In our research, we focus on ILs that feature acrylate or methacrylate functional groups for this particular purpose. Polymerization and reactions such as the alkene hydrothiolation are candidates to link the ILs to a covalent network.

A common method for the preparation of ammonium ionic liquids is a reaction pathway with two steps. In a first step, the tertiary amine is converted in a quaternary ammonium salt by Menshutkin reaction [13,14]. Within the second step, the halogen anion is exchanged for an organic anion, e.g., PF_6_, BF_4_ or TFSI [15,16,17]. Preparation of ionic liquids starting from the polymerizable amines 2-(diethylamino)ethyl methacrylate (DEAEMA) and 2-(diethylamino)ethyl acrylate (DEAEA) is rarely described in the literature [18]. However, there are few reports on the synthesis of the halogen precursors by Menshutkin reaction [13,14]. It is known that the turnover of DEAEA to the desired precursor lowers drastically when the quaternization reaction is performed with alkyl bromides containing more than two carbon atoms. Regarding to the literature, yields decrease from 62% (DEAEA + C_2_H_5_Br) [19] to 13% (DEAEA + C_4_H_9_Br) [19] and 2.1% (DEAEA + C_6_H_9_Br) [18]. This is consistent with the general trend that is found for the reaction rate constant of *S_N_*2 reactions which decreases with increasing size of the alkylating agent [20]. In terms of reaction mechanism, the Menshutkin reaction can be seen as a special type of *S_N_*2 reaction [21]. In comparison to other *S_N_*2 type reactions, the reactions rate is promoted by increasing solvent polarity [21]. Synthesis of similar ionic liquids starting from the methyl substituted amines 2-(dimethylamino)ethyl methacrylate (DMAEMA) or 2-(dimethylamino)ethyl acrylate (DMAEA) were described by several authors [18,22]. In the literature, alkyl bromides and acetone or chloroform were used as reagent and solvent, respectively, thus resulting in high yields for the precursors, e.g., 89% for [C_4_N_MA,11_]Br [22] and 82.6% for [C_6_N_MA,11_]Br [18]. In contrast to the earlier mentioned precursors, the yields for the quaternization of DMAEMA and DMAEA are still reasonable for alkyl substituents with more than two carbon atoms.

For application in energy storage systems the relationship between structure variations and fundamental properties such as glass transition temperature, viscosity and ionic conductivity are of great interest. Two common models for data interpretation are the Arrhenius equation (1) and the Vogel–Fulcher–Tammann (VFT) Equation (2)

(1) $$\eta =A_{\eta }e^{E_{\eta }/RT};\;\sigma =A_{\sigma }e^{-E_{\sigma }/RT}$$

(2) $$\eta =A_{\eta }e^{k_{\eta }/\left(T-T_{0}\right)};\;\sigma =A_{\sigma }e^{-k_{\sigma }/\left(T-T_{0}\right)}$$

with *A_η_* and *A_σ_* as the viscosity and conductivity at infinite temperature, *E_η_* and *E_σ_* as flow and conductivity activation energies, the ideal gas constant *R*, the temperature *T*, *k_η_* and *k_σ_* as constants and *T*_0_ as ideal glass transition temperature [23]. In the literature the ideal parameter *T*_0_ is described as temperature where free volume disappears so that cooperative movement of chain segments, as well as motion related phenomena such as diffusion or migration of molecules and ions becomes impossible [23]. Due to kinetic reasons, this state cannot be reached within a limited time scale and *T*_0_ is found to be lower than experimentally measured values for *T_g_* [23]. Correlation of the different fundamental properties offers further possibilities for analysis and interpretation of the experimental results. Scaling the Arrhenius plot to experimentally measured *T_g_* values leads to the Arrhenius plot of fragilities or so-called Angell plot. Thereby, glass builders are classified as *strong* or *fragile*. The liquid structure of *fragile* materials responses very sensitive to temperature changes, which is associated with a relative narrow glass transition temperature range. *Strong* glass builders response in an opposite way [24,25]. Another interpretation is made by the Walden plot. In 1906 Paul Walden released his underlying empirically found relationship, that the product of viscosity of a solvent and its molar conductivity is constant [26]. Within the plot, experimental data are related to a line of an ideal electrolyte solution, which commonly is a 1-molar KCl solution. In the field of ionic liquids, the position of the ILs line is compared to the ideal line to classify the ILs in *superionic* liquids, *good* ILs and *poor* ILs concerning the decoupling of one charge carrier from the viscosity determined structure [27]. In the case of *superionic* the ILs line is located above the ideal line, which leads to the interpretation that one charge carrier species is partially decoupled from the viscous bulk. Slightly below and directly on the ideal line, ILs are classified as *good* ionic liquids featured by a quasi-lattice state and a low ionicity. The more the ILs line is below the ideal line, the more this state is shifted to direct ion pairing and higher ionicities [27].

In this study, we focus on the systematic preparation and characterization of polymerizable ionic liquids starting from DEAEMA and DEAEA. First, we describe the optimization of the reaction conditions to achieve almost quantitative precursor yields even for steric demanding alkyl chains and the adapted anion exchange. Subsequently, the characterization of the IL products is presented with respect to an application of the ionic liquids in energy storage systems. Structure-property relationships of the monomeric ILs are analyzed and their adaptability on polymerized ionic liquids are discussed.

## 2. Results and Discussion

### 2.1. Synthesis

**Figure 1:**
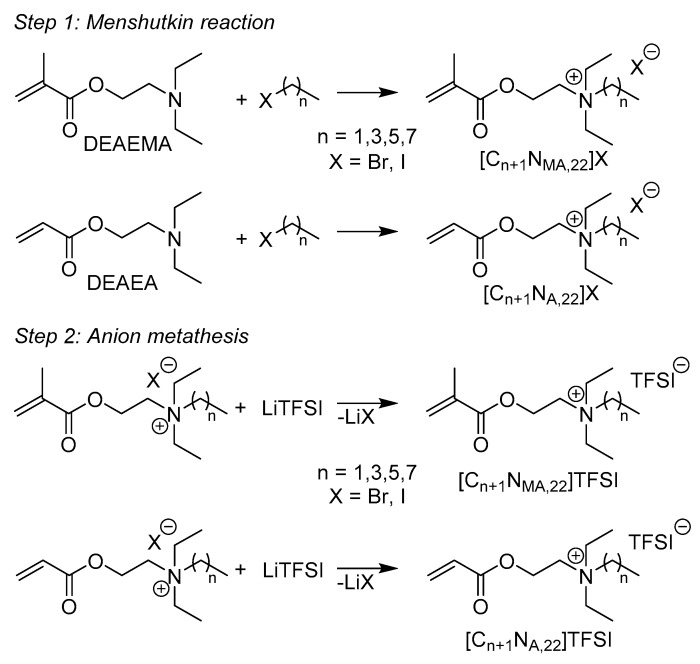
Reaction scheme for the preparation of the IL and notation.

Eight new ionic liquids were prepared in a two-step synthesis, shown in Figure 1. In the first step the amines DEAEMA and DEAEA were converted into quaternary ammonium bromide or iodide salts using the Menshutkin reaction [13,14]. In a following metathesis reaction, the halogen anions were exchanged by the organic anion bis(trifluoromethanesulfonyl)imide (TFSI).

Compared to the dimethyl substituted ammonium ionic liquids known from literature [11,22], two ethyl substituents are attached to the nitrogen atom in DEAEMA and DEAEA. This variation is associated with a higher steric hindrance at the amine function. We found that the yields of the quaternization reaction to bromide precursors are much lower when comparable reaction conditions from the literature were used with DEAEMA and DEAEA as starting materials. With increasing lengths of alkyl chains, the yields dropped drastically and fell below a reasonable level for C_4_-C_8_ rests. In order to achieve the quaternary ammonium salts in high yields, different reaction conditions were applied. Table 1 gives an overview of selected reactions. To avoid declining yields with increasing chain lengths, the reaction time and temperature were increased, thus leading to a partial degradation of the double bonds. Referring to Daoudi et al., the reaction of DEAEMA with *n*BuBr in dichloromethane was supposed to proceed almost quantitatively when the mixture was heated 8 h under reflux [28]. Applying similar conditions (16 h of reflux), the reaction failed and no product was obtained. Yields were significantly enhanced by the combination of alkyl iodides and acetone as reactants and solvent, respectively, as well as by the usage of acetonitrile as solvent when alkyl bromides were used. In *S_N_*2 type reactions iodide is the better leaving group compared to bromide, due to its lower basicity. Solvents with higher polarity affect the Menshutkin reaction positively [21]. Accordingly, the combination of acetonitrile as solvent and alkyl iodides as reactants led to almost quantitative yields, while the excess of alkyl halides was reduced.

All synthesized ammonium bromide compounds are strongly hygroscopic and feature a high solubility in water. Hence, using bromide intermediates, the anions exchange was executed in an aqueous solution from which the desired product precipitates. The synthesized iodide intermediates show a significantly lower hygroscopy and are therefore easier to handle under ambient conditions. A drawback compared to bromides is the accompanied decrease in water solubility, which makes a metathesis in pure water difficult for most of the precursor iodides. Instead the meth(acrylate) iodides were dissolved in acetone and added dropwise to an aqueous LiTFSI solution from which the IL phase precipitated immediately. The attempt to remove undesirable residues by extraction of the ionic liquids with chloroform and washing the organic phase with water points out to be impractical. The solubility of the ILs in chloroform is strongly limited and two organic phases appeared. The high viscosity and adhesion on the glass surface of the ionic liquid enriched phases, makes an effective separation from the aqueous phase almost impossible, when a separation funnel is used. In contrast, washing the IL monomers directly several times with purified water and decanting the aqueous phase, removed the remaining acetone and lithium halide salts from the products in an easy and effective manner. Table 2 gives an overview of the achieved yields concerning the two-step synthesis with ammonium iodide salts as intermediate products.

To accelerate the preparation of the ILs, a one-pot synthesis for [C_2_N_MA,22_]TFSI was carried out, by way of example. Without optimization of reaction conditions, a total yield of 66% was achieved, making the one-pot synthesis easily accessible to industrial scales.

It was found in the study that for long-term-storage the ionic liquids must be handled with an inhibitor to avoid any polymerization. An appropriate inhibitor which can be used when oxygen is present in the sample is MEHQ (Hydroquinone monomethyl ether) [29].

### 2.2. Density

**Figure 2:**
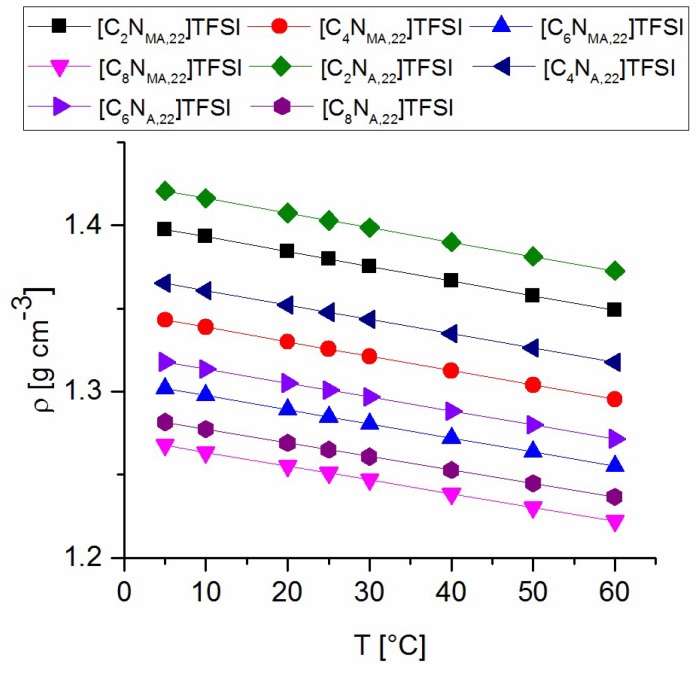
Density values of the synthesized TFSI-Ionic Liquids.

Density values of all prepared TFSI-ionic liquids were measured between 5 °C and 60 °C. The resulting data are plotted in Figure 2 and fitted to a linear function *y* = *m* × *x* + *n*. The calculated parameters are presented in Table 3. Density values of the acrylates are higher than the values of the methacrylate ILs with respect to the same alkyl chain lengths. With an increasing length of the attached alkyl group, density values decrease. Longer chains and the variation from acrylate to methacrylate groups lead to increased bulkiness and steric demand of the molecules, which is expressed in lower density values.

As the linear fitting shows, the lines are almost parallel. The slopes indicate a tendency to flatten with increasing alkyl chain length. The parameter *n* shows the differences between the calculated densities at 0 °C, which decrease with increasing chain length towards smaller density values. Density values of new ionic liquids of the same homologous series can be estimated on basis of the presented interpretation.

### 2.3. Thermal Analysis

DSC analysis was applied to the halogen salts and TFSI-ionic liquids. Data of the melting points result from a single measurement. For glass transition temperatures the probes were heated twice from −150 °C to 180 °C with 10 K min^−1^. The given *T_g_* values are the values of detected inflection points of the first occurring glass transition (*T_g_*(*L*)) for each substance at the first heating curve.

**Figure 3:**
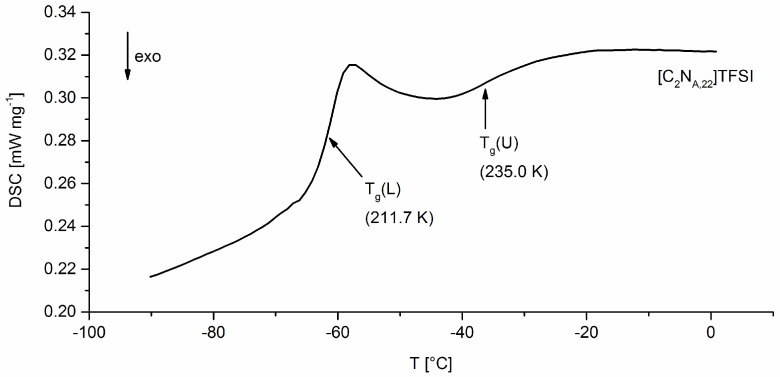
DSC heating curve of [C_2_N_A,22_]TFSI which stands exemplary for all synthesized ILs.

As shown in Figure 3, two second order phase transitions, *T_g_*(*L*) and *T_g_*(*U*), appear in DSC analysis. *T_g_*(*L*) refers to the conventional glass transition and is therefore labeled as *T_g_* in the further text. The appearance of *T_g_*(*U*) might be associated with semi-crystalline, smectic or para-crystalline phases as discussed in literature for semi-crystalline polymers [30].

Table 4 shows the resulting data of DSC analysis and gives two different values for *T_g_* (*T_g_*(*L*)). The *T*_*g*,1_ value was measured two weeks after synthesis in a single probe analysis. The second value, *T*_*g*,2_, is the average glass transition temperature of three probes, which were of the same batch, but stored eight weeks before DSC analysis was applied. All freshly synthesized ionic liquids were diluted in acetone (50 wt%) and stored at −40 °C. Before any analysis, the acetone was completely removed under vacuum at room temperature within an argon glove box.

The data indicate that the intermediates melting points decrease with increasing length of the introduced alkyl chains, except [C_4_N_MA,22_]I. The acrylate iodides show lower melting points than the methacrylate iodides. The studied bromid salt intermediates feature higher melting points than the analogous iodides.

The glass transition temperatures of the ionic liquids tend to decrease with increasing alkyl length. It is remarkable that the *T*_*g*,2_ values are in average 4.3 K higher than *T*_*g*,1_ values. There is evidence that this observation is due to partial polymerization processes, although the ionic liquids are diluted in acetone (50 wt%) and stored at −40 °C. The process of double bond degradation is also seen on the morphology of the ionic liquids after the acetone is removed. In contrast to freshly prepared ionic liquids, the probes start to thread after storing them several weeks. For analysis in Arrhenius plot of fragilities *T*_*g*,1_ values are used due to the evidence that these values are less influenced by autopolymerization.

### 2.4. Rheological Behavior

**Figure 4:**
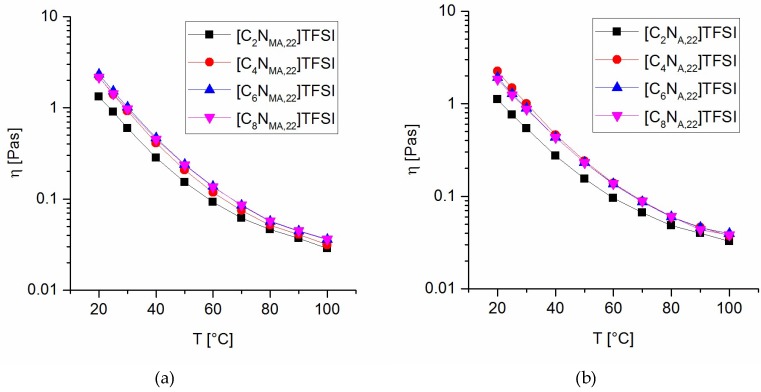
Viscosity values at various temperatures for methacrylate ILs (**a**) and acrylate ILs (**b**).

The viscosity of the ionic liquid monomers was studied in two manners. First, a constant temperature of 20 °C and varying shear rates from 1 s^−1^ to 250 s^−1^ were applied, resulting in shear rate independent viscosity for all the prepared ILs (within 1 s^−1^ to 250 s^−1^). Second, a temperature program from 20 °C to 100 °C at a constant shear rate of 100 s^−1^ was applied. The dependence of the viscosity values on the temperature is given in Figure 4.

The results reveal that the ethyl-substituted ILs have the lowest viscosity values within the studied temperature range. Butyl- to octyl-substituted derivates show low divergence in the viscosity values and temperature dependence and thus, no correlation between the chain lengths and the viscosity values was found for these derivates. In general, the methacrylate ionic liquids tend to higher viscosities than the analogous acrylates at same temperatures. The data indicate that bulky groups within the ILs promote higher viscosity values. Phenomena such as entanglements increase the internal friction and are most likely to be causal for this observation. Tokuda et al. found the same property-structure relationship for imidazolium TFSI ionic liquids and correlated it to the influence on the ionic self-diffusivity [31].

### 2.5. Electrochemical Characterization

**Figure 5:**
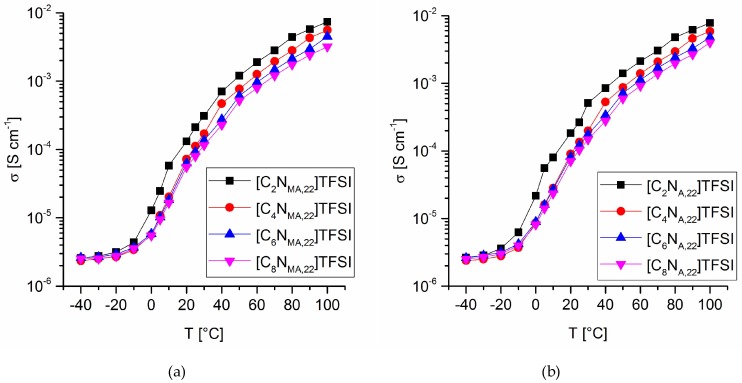
Ionic conductivity values at various temperatures for methacrylate ILs (**a**) and acrylate ILs (**b**).

The ionic conductivity of the TFSI ionic liquids was calculated from the corresponding impedance spectra. The closed, airtight measuring cells for the liquid monomers were assembled inside an argon-purged glovebox (O_2_ and H_2_O < 0.5 ppm). Electrochemical impedance spectroscopy experiments were done at temperatures between −40 °C and 100 °C. During the measurements, the cells were located inside an oven. After the adjusted temperatures reached the desired values, further 20 min were given to the system before measurement to ensure a steady state. Voltage for potentiostatic impedance spectroscopy was set to 0.000 V. Amplitudes of 10 mV were applied with varying frequencies within the range of 100 Hz to 1 MHz. Figure 5 shows the temperature dependency of the ionic conductivity values. From 100 °C to 10 °C, the conductivity values decrease logarithmically. Below 5 °C there is an abrupt flatten of the conductivity curves, which indicates a phase transition within the given temperature range. This is consistent with the appearance of the upper glass transition temperature, *T_g_*(*U*), in DSC. Due to the limitation of ionic mobility, the conductivity values strongly approach *σ*(*Tg*) with onset of the upper phase transition.

There is a significant impact of the structural modifications on the ionic conductivity, showing a straight structure-property relationship. It was found, that increasing chain lengths of the introduced alkyl group correlate with lower ionic conductivities. Furthermore, the acrylates show higher conductivity values than the methacrylate derivates at the same alkyl chain lengths and temperatures. The conductivities at 25 °C ranging from 0.264 mS cm^−1^ ([C_2_N_A,22_]TFSI) down to 0.080 mS cm^−1^ ([C_8_N_MA,22_]TFSI), as given in Table 5.

### 2.6. Analysis of Structure-Property Relationships

The variation between acrylate and methacrylate terminal function as well as the length of the introduced alkyl chain significantly affect the ionic liquids physicochemical properties. The measured densities decrease with increasing chain lengths and steric demand of the side chains, to remain the same trends found for the ionic conductivity on the molar conductivity. Ionic conductivity values decrease and viscosity values tend to increase with increasing steric demand of the attached side chains. Similar influences of structural variations on density, rheological behavior and conductivity were reported in the literature for imidazolium TFSI ILs, indicating a general trend for structure-property relationship of ionic liquids [31].

Considering the influence of the attached alkyl chains on the conductivity and viscosity values in Table 5, it turns out that the step from C_2_ to C_4_ chains is accompanied with a more pronounced property change than any other step in chain prolongation. For both homologous series, acrylates and methacrylates, the ionic conductivity drops almost 50% from C_2_ to C_4_ chains, whereas the step from C_4_ to C_8_ rests leads to a decrease in the order of 25% to 30%. The trend found for the viscosity values shows a similar behavior. There is a significant increase in viscosity when going from C_2_ to C_4_ groups. From C_4_ to C_8_ side chains there is no straight property-structure relationship identifiable and the occurring variations of viscosity values are much less pronounced than caused by the step from C_2_ to C_4_.

Since all studied ionic liquids are featured by the same anion, the variance in the chemico-physical values is primarily driven by the structural differences of the cations. On one side, longer and bulkier sidechains lead to a higher steric demand of the molecules on the molecular level, thus resulting in lower densities. On the other side, higher densities cause stronger intermolecular forces, concerning anion-cation interactions as well as attractive forces between the organic cations, such as aliphatic π-interactions. As expected from this consideration, it was found that the glass transition temperatures tend to decrease with increasing length of the substituted alkyl groups due to higher free volumes, but contradicts the influence of the methyl group at the double bond. Table 6 shows that the studied acrylate ILs feature lower glass transition temperatures than the respective methacrylates. Therefore, it is assumed that the higher steric freedom of the acrylate function without attached methyl group has a more dominant influence on *T_g_* than stronger electrostatic interactions between the cations.

The introduced alkyl chains are accompanied with an electron donating inductive effect. Longer alkyl chains will lead to a reduction of charge density at the cationic center. Thus, induce a more independent mobility of cations and anions and result in a higher ionic conductivity. Experimentally the opposite observation was made on the ionic conductivity. Therefore, it is stated that the electrostatic effect of alkyl chains has a negligible impact on the ionic conductivity. The adverse influence of longer chains on the cations mobility is most likely to limit the conductivity in impedance spectroscopy experiments.

**Figure 6:**
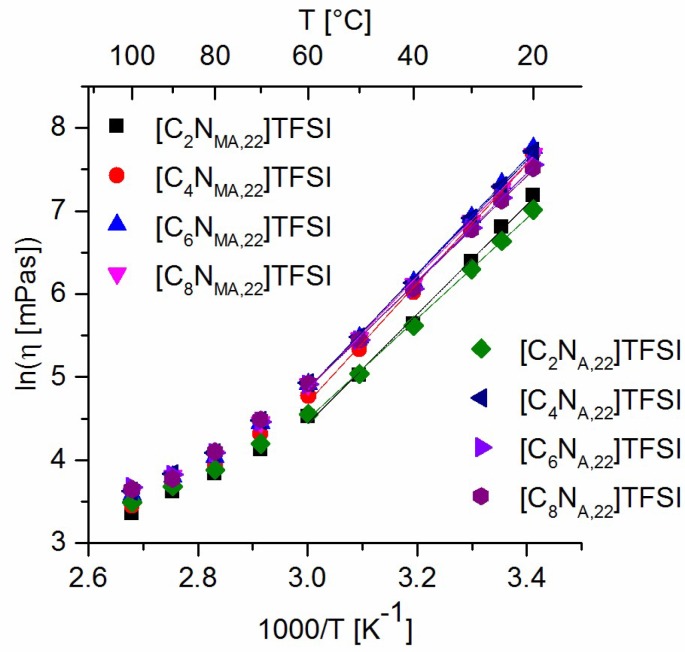
Arrhenius fit of viscosity values.

The influence of the substituents steric demand on the cations mobility can be derived by the comparison of viscosity values of the different ILs. Figure 6 shows the empirical analysis of the viscosity values in terms of an Arrhenius plot (equation 1). The data between 20 °C and 60 °C were linear fitted and the flow activation energy was calculated within this temperature range. The results are given in Table 7 and show the same behavior as discussed for the viscosity values.

**Figure 7:**
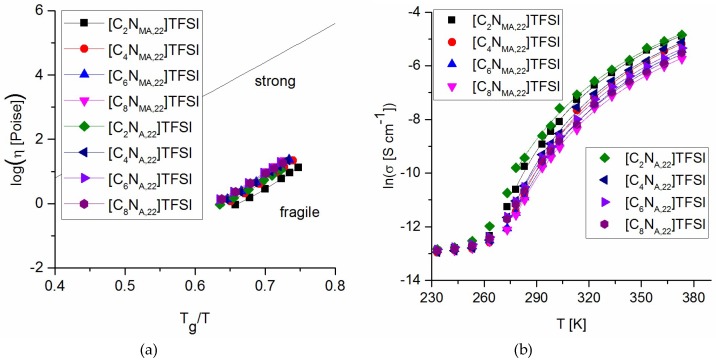
(**a**) Arrhenius plot of fragilities; (**b**) VFT fit of ionic conductivity values.

With respect to the experimentally measured glass transition temperatures and the viscosities, Figure 7a represents the Arrhenius plot of fragility. Due to the analysis, all studied ionic liquids are classified as *fragile*. They react sensitive to thermal excitation and thus show a narrow temperature range of glass transition.

Empirical description of ionic conductivity values is done by VFT fit shown in Figure 7b (Equation (2)). The analysis was initialized by iterative non-linear least squares to give values for *T*_0_ (Table 6). Due to this procedure, the ionic conductivity data fit well to the VFT equation in a temperature range between 5 °C and 100 °C. Below this range, conductivity values approach the conductivity at *T_g_*. As expected, *T*_0_ values are below the measured *T_g_* values and *T_g_*/*T*_0_ is within the same range as found for other TFSI-ionic liquids in the literature [23].

**Figure 8:**
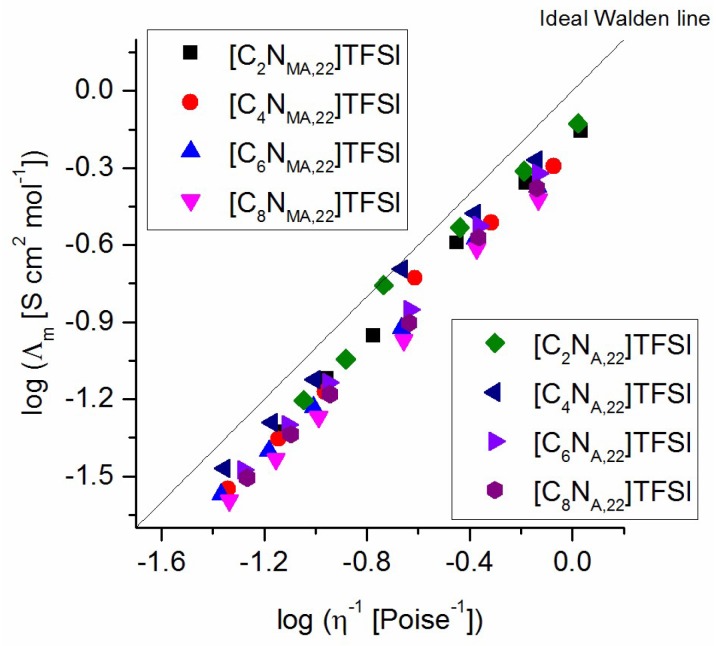
Walden plot for the studied TFSI ionic liquids.

The correlation of molar conductivity and viscosity of the studied ionic liquids is plotted in a Walden plot. Figure 8 shows the resulting linear relationship, which indicates a completely dissociation of all ions in the system. The data for all ionic liquids are located slightly below the ideal Walden line, which implies that the charge carriers are coupled to the viscosity determining structure. In terms of the Walden plot, all products are classified as *good* ionic liquids. The lines of the studied ILs are very close to each other, which shows that there are only small differences in ionicities between the ILs. This supports the already discussed assumption that the strength of cation-anion interactions is not the causal driving force for the found structure-property relationships.

Regarding the application of the studied ionic liquids in electrolyte systems, acrylates with short alkyl chains seem to outperform methacrylates and more bulky substituents due to the lower viscosity and higher conductivity. Although they found similar behaviors for the ionic liquids, Zhang et al. showed that the polymer of undoped [C_7_N_MA,11_]TFSI reveals a higher ionic conductivity than the [C_4_N_MA,11_]TFSI polymer [22]. Therefore, the property-relationships of the pure ILs cannot be directly transferred on their polymers. Further, additional material properties such as strength and brittleness of the polymers and the influences of conducting salt and additives must be regarded separately for particular applications.

In pretests, the monomers were successfully polymerized. For example, compound [C_2_N_MA,22_]TFSI can be polymerized via UV induced bulk polymerization with 1 mol-% initiator (Irgacure 651) resulting in hard and brittle glasses. However, a broad range of parameters influences the polymeric matters properties. Thus, there are many parameters influencing the polymers properties starting with the general type of chosen polymerization method, type of initiator, initiator concentration and so on. Additionally, a huge toolbox of analytical methods for characterization, such as DSC, electrochemical impedance spectroscopy, cyclic voltammetry or size-exclusion chromatography, is available. With respect to an application in energy storage systems further parameters such as the type of applied conducting salt, salt concentration or additives come in addition. All these effects influence the structure and properties of the polymers. The presented results are focusing on the synthesis and characterization of the polymerizable monomers. In respect of its huge scope, the study of the polymerized ionic liquid materials will soon be presented separately.

## 3. Conclusions

In this study, we have synthesized and characterized eight new ionic liquids to improve the understanding of how structural changes affect the physicochemical properties of ionic liquids. Reaction conditions were optimized to obtain almost quantitative yields for the yield limiting quaternization reaction. The study reveals that an increasing side chain length at the ammonium moiety as well as and methyl group at the acrylate function accompany with increasing viscosity and decreasing ionic conductivity values. At 25 °C the ionic conductivity of the studied ionic liquids varies between 0.264 mS cm^−1^ for [C_2_N_A,22_]TFSI and 0.080 mS cm^−1^ and viscosity values between 0.762 Pa s [C_2_N_A,22_]TFSI and 1.522 Pa s [C_6_N_MA,22_]TFSI were found. Ionicity of the investigated ionic liquids shows low diversity in Walden plot. It is therefore assumed that the structure-property relationships of tetraalkylated ammonium ionic liquids are mainly driven by the steric properties of the cation on molecular level. It was found that the examined ionic liquids tend to autopolymerize when storing them diluted in acetone (50 wt%) at −40 °C. For the permanent usage at ambient conditions and elevated temperatures an inhibitor system will be required to prevent degradation of the double bonds. The data reveal that the polymerizable ionic liquids with short sidechains have favorable properties for an application in ionic conducting materials. Nevertheless, the literature indicate that the structure-property relationships found for the monomeric ionic liquids cannot be transferred on their polymers without further investigation.

## 4. Experimental Section

### 4.1. Materials

2-(Diethylamino)ethyl methacrylate (99%), 2-(diethylamino)ethyl acrylate (95%), bromoethane (98%), 1-bromobutane (99%), 1-bromohexane (≥99%), phenothiazine (≥98%) and bis(trifluoromethane)sulfonimide lithium salt (99%) were purchased from Sigma-Aldrich (Munich, Germany). Iodoethane (98%), 1-iodobutane (98%), 1-iodohexane (98%), 1-iodooctane (98%) and molecular sieves (3°Å, 8-12 mesh) were received from Acros Organics (Geel, Belgium). Acetone (≥98.8%) and diethyl ether (anhydrous) were purchased from Fisher Scientific (Schwerte, Germany). Water (HPLC grade) and acetonitrile (≥99.7%; HPLC grade) were purchased from Carl Roth (Karlsruhe, Germany) and Alfa Aesar (Karlsruhe, Germany), respectively. All chemicals were used as received.

### 4.2. Measurements

NMR spectra were recorded using an *Avance III HD 500 MHz* spectrometer (Bruker, Germany) and ATR-FTIR spectra with a *Vertex 70* (Bruker, Germany). Dynamic viscosities of the ILs were measured with a *Gemini HR Nano* rheometer (Bohlin, UK) and cone-plate system (CP4/40). For impedance spectroscopy a *Zahner Zennium* potentiostat (Zahner, Germany) and *TSC 1600 Closed* measurement cells (rhd, Germany) located inside a *SH-261* oven (Espec, USA) were applied. The water content of the ILs was measured by Karl Fischer-titration with a *KF Coulometer 831* (Metrohm, Germany) coupled with a *KF Thermoprep 860* oven (Metrohm, Germany) at 180 °C and flow rate of 150 mL min^−1^. Melting points and glass transition temperatures were measured by DSC analysis using a *DSC 204 F1 Phoenix* (Netzsch, Germany) with temperature programs between −150 °C and 180 °C and heating rates of 10 K min^−1^. Elemental analysis was carried out with a *vario MICRO* (Elementar, Germany) and a *SARTORIUS M2P* was used as analytical balance. Densities of ionic liquids were measured with a *DMA 4500 M* (Anton Paar, Austria) between 0.0 °C and 60.0 °C.

### 4.3. Synthesis

All solid products are stored in amber glass flasks inside an argon-filled glovebox. All TFSI ionic liquids are diluted to 50 wt% in acetone under argon and stored under exclusion of light at −30 °C. After removing the acetone, the ILs show less than 10 ppm water in Karl Fischer-titration. For synthesis and washing of ionic liquids deionized water (HPLC grade) was used without exceptions.

#### *N,N,N*-Triethyl-*N*-[2-(methacryloyloxy)ethyl]ammonium bromide (**[C_2_N_MA,22_]Br**)

A mixture of 2-(diethylamino)ethyl methacrylate (DEAEMA, 10.99 g, 59.32 mmol) and a excess of bromoethane (9.24 g, 84.84 mmol) in 30 mL acetone was stirred under argon-oxygen atmosphere (80:20% *v*/*v*) at 47 °C for 6 days and under exclusion of UV irradiation. All volatile components were removed under reduced pressure. The precipitate was washed with ethyl ether and dried under vacuum for 10 h. The product was received as a white powder (10.61 g, 60.8% yield). M.p.: 111.2 °C; ^1^H NMR (500 MHz, CDCl_3_): δ (ppm) 1.45 (t, 9H, CH_3_); 1.95 (s, 3H, CH_3_); 3.64 (q, 6H, CH_2_); 3.97 (t, 2H, CH_2_);); 4.76 (t, 2H, CH_2_); 5.68 (s, 1H, CH_2_); 6.12 (s, 1H, CH_2_); ^13^C NMR (125 MHz, CDCl_3_): δ (ppm) 8.3 (CH_3_); 18.3 (CH_2_); 54.4 (CH_2_); 56.0 (CH_2_); 57.8 (CH_2_); 127.5 (CH_2_); 135.1 (C); 166.4 (C=O); IR (cm^−1^): 2979 ν(C-H); 1717 ν(C=O); 1638 ν(C=C); 1479 δ_as_(CH_2_); 1390 δ_s_(CH_3_); 1320 δ(CH_2_); 1298 ν(C-O); 1168 ν(C-O); 1002 δ(CH_2_); 947 δ(C-H); 784 δ(CH_2_);

#### *N*-Butyl-*N,N*-diethyl-*N*-[2-(methacryloyloxy)ethyl]ammonium bromide (**[C_4_N_MA,22_]Br**)

A mixture of 2-(diethylamino)ethyl methacrylate (DEAEMA, 11.12 g, 60.00 mmol) and a excess of 1-bromobutane (11.51 g, 84.00 mmol) in 30 mL acetone was stirred under argon-oxygen atmosphere (80:20% *v*/*v*) under reflux for 11 days and under exclusion of UV irradiation. All volatile components were removed under reduced pressure. The precipitate was washed with ethyl ether and dried under vacuum for 24 h. The product was received as a white powder (9.49 g, 49.1% yield). M.p.:107.9 °C; ^1^H NMR (500 MHz, CDCl_3_): δ (ppm) 1.02 (t, 3H, CH_3_); 1.47 (m, 8H, CH_3_, CH_2_); 1.76 (m, 2H, CH_2_); 1.96 (s, 3H, CH_3_); 3.44 (m, 2H, CH_2_); 3.67 (q, 4H, CH_2_); 4.01 (t, 2H, CH_2_); 4.69 (t, 2H, CH_2_); 5.70 (s, 1H, CH_2_); 6.14 (s, 1H, CH_2_); ^13^C NMR (125 MHz, CDCl_3_): δ (ppm) 8.4 (CH_3_); 13.7 (CH_3_); 18.3 (CH_3_); 19.8 (CH_2_); 24.1 (CH_2_); 55.4 (CH_2_); 56.6 (CH_2_); 57.8 (CH_2_); 58.9 (CH_2_); 127.5 (CH_2_); 135.1 (C); 166.5 (C=O); IR (cm^−1^): 2963 ν(C-H); 1721 ν(C=O); 1640 ν(C=C); 1457 δ_as_(CH_2_); 1399 δ_s_(CH_3_); 1300 ν(C-O); 1159 ν(C-O); 1013 δ(CH_2_); 934 δ(C-H); 788 δ(CH_2_); Elemental analysis calcd (%) for C_14_H_28_BrNO_2_: C, 52.18; H, 8.76; N, 4.35. Found (%): C, 51.59; H, 8.49; N, 4.41.

#### *N,N*-Diethyl-*N*-hexyl-*N*-[2-(methacryloyloxy)ethyl]ammonium bromide (**[C_6_N_MA,22_]Br**)

A mixture of 2-(diethylamino)ethyl methacrylate (DEAEMA, 5.59 g, 30.02 mmol) and a excess of 1-bromohexane (6.93 g, 42.00 mmol) in 15 mL acetonitrile was stirred under argon-oxygen atmosphere (80:20% *v*/*v*) at 56 °C for 6 days and under exclusion of UV irradiation. All volatile components were removed under reduced pressure. The precipitate was washed with ethyl ether and dried under vacuum. The product was received as a white powder (5.67 g, 53.9% yield). M.p.:102.7 °C; ^1^H NMR (500 MHz, CDCl_3_): δ (ppm) 0.87 (t, 3H, CH_3_); 1.33 (m, 6H, CH_2_); 1.43 (t, 6H, CH_3_); 1.74 (m, 2H, CH_2_); 1.93 (s, 3H, CH_3_); 3.40 (m, 2H, CH_2_); 3.64 (q, 4H, CH_2_); 3.97 (t, 2H, CH_2_); 4.65 (t, 2H, CH_2_); 5.66 (s, 1H, CH_2_); 6.11 (s, 1H, CH_2_); ^13^C NMR (125 MHz, CDCl_3_): δ (ppm) 8.3 (CH_3_); 16.9 (CH_3_); 18.3 (CH_3_); 22.2 (CH_2_); 22.4 (CH_2_); 26.1 (CH_2_); 31.2 (CH_2_); 55.0 (CH_2_); 56.6 (CH_2_); 57.9 (CH_2_); 59.0 (CH_2_); 127.4 (CH_2_); 135.1 (C); 166.4 (C=O); IR (cm^−1^): 2928 ν(C-H); 1717 ν(C=O); 1640 ν(C=C); 1446 δ_as_(CH_2_); 1399 δ_s_(CH_3_); 1298 ν(C-O); 1163 ν(C-O); 1009 δ(CH_2_); 945 δ(C-H); 813 δ(CH_2_); Elemental analysis calcd (%) for C_16_H_32_BrNO_2_: C, 54.85; H, 9.21; N, 4.00. Found (%): C, 54.73; H, 9.17; N, 4.01.

#### *N,N,N*-Triethyl-*N*-[2-(methacryloyloxy)ethyl]ammonium iodide (**[C_2_N_MA,22_]I**)

A mixture of 2-(diethylamino)ethyl methacrylate (DEAEMA, 27.789 g, 150.0 mmol), a slight excess of iodoethane (25.735 g, 165.0 mmol) and phenothiazine (0.6 g, 3.0 mmol) as inhibitor in 15 mL acetonitrile was stirred under argon atmosphere at 45 °C for 24 h and under exclusion of UV irradiation. All volatile components were removed under reduced pressure. The precipitate was washed with ethyl ether and dried under vacuum. The product was received as a white powder (49.32 g, 96.4% yield). M.p.: 102.7 °C; ^1^H NMR (500 MHz, acetone-D6): δ (ppm) 1.46 (t, 9H, CH_3_); 1.96 (s, 3H, CH_3_); 3.73 (q, 6H, CH_2_); 4.02 (t, 2H, CH_2_); 4.72 (t, 2H, CH_2_); 5.74 (s, 1H, CH_2_); 6.14 (s, 1H, CH_2_); ^13^C NMR (125 MHz, acetone-D6): δ (ppm) 7.6 (CH_3_); 17.6 (CH_2_); 53.9 (CH_2_); 55.6 (CH_2_); 58.0 (CH_2_); 126.1 (CH_2_); 135.8 (C); 166.1 (C=O); IR (cm^−1^): 2974 ν(C-H); 1719 ν(C=O); 1635 ν(C=C); 1457 δ_as_(CH_2_); 1397 δ_s_(CH_3_); 1300 ν(C-O); 1165 ν(C-O); 1000 δ(CH_2_); 949 δ(C-H); 782 δ(CH_2_); Elemental analysis calcd (%) for C_12_H_24_INO_2_: C, 42.24; H, 7.09; N, 4.10. Found (%): C, 42.24; H, 7.11; N, 4.10.

#### *N*-Butyl-*N,N*-diethyl-*N*-[2-(methacryloyloxy)ethyl]ammonium iodide (**[C_4_N_MA,22_]I**)

A mixture of 2-(diethylamino)ethyl methacrylate (DEAEMA, 27.789 g, 150.0 mmol), a slight excess of 1-iodobutane (30.363 g, 165.0 mmol) and phenothiazine (0.6 g, 3.0 mmol) as inhibitor in 15 mL acetonitrile was stirred under argon atmosphere at 60 °C for 3 days and under exclusion of UV irradiation. All volatile components were removed under reduced pressure. The precipitate was washed with ethyl ether and dried under vacuum. The product was received as a slightly yellowish powder (54.02 g, 97.5% yield). M.p.: 116.1 °C; ^1^H NMR (500 MHz, acetone-D6): δ (ppm) 1.02 (t, 3H, CH_3_); 1.49 (m, 8H, CH_3_, CH_2_); 1.90 (m, 2H, CH_2_); 1.96 (t, 3H, CH_3_); 3.62 (m, 2H, CH_2_); 3.75 (q, 4H, CH_2_); 4.00 (m, 2H, CH_2_); 4.72 (m, 2H, CH_2_); 5.75 (t, 1H, CH_2_); 6.15 (s, 1H, CH_2_); ^13^C NMR (125 MHz, acetone-D6): δ (ppm) 7.4 (CH_3_); 13.0 (CH_3_); 17.5 (CH_3_); 19.5 (CH_2_); 23.7 (CH_2_); 54.4 (CH_2_); 56.0 (CH_2_); 57.9 (CH_2_); 58.2 (CH_2_); 126.0 (CH_2_); 135.8 (C); 166.0 (C=O); IR (cm^−1^): 2963 ν(C-H); 1724 ν(C=O); 1640 ν(C=C); 1458 δ_as_(CH_2_); 1401 δ_s_(CH_3_); 1300 ν(C-O); 1163 ν(C-O); 1013 δ(CH_2_); 960 δ(C-H); 813 δ(CH_2_); Elemental analysis calcd (%) for C_14_H_28_INO_2_: C, 45.53; H, 7.64; N, 3.79. Found (%): C, 45.48; H, 7.64; N, 3.73.

#### *N,N*-Diethyl-*N*-hexyl-*N*-[2-(methacryloyloxy)ethyl]ammonium iodide (**[C_6_N_MA,22_]I**)

A mixture of 2-(diethylamino)ethyl methacrylate (DEAEMA, 27.789 g, 150.0 mmol), a slight excess of 1-iodohexane (34.992 g, 165.0 mmol) and phenothiazine (0.6 g, 3.0 mmol) as inhibitor in 15 mL acetonitrile was stirred under argon atmosphere at 60 °C for 3 days and under exclusion of UV irradiation. All volatile components were removed under reduced pressure. The precipitate was washed with ethyl ether and dried under vacuum. The product was received as a white powder (57.18 g, 95.9% yield). M.p.: 95.7 °C; ^1^H NMR (500 MHz, acetone-D6): δ (ppm) 0.91 (t, 3H, CH_3_); 1.39 (m, 6H, CH_2_); 1.49 (t, 6H, CH_3_); 1.92 (m, 2H, CH_2_); 2.00 (s, 3H, CH_3_); 3.62 (m, 2H, CH_2_); 3.75 (q, 4H, CH_2_); 4.01 (m, 2H, CH_2_); 4.72 (s, 2H, CH_2_); 5.75 (s, 1H, CH_2_); 6.15 (s, 1H, CH_2_); ^13^C NMR (125 MHz, acetone-D6): δ (ppm) 7.4 (CH_3_); 13.3 (CH_3_); 17.5 (CH_3_); 21.7 (CH_2_); 22.2 (CH_2_); 25.8 (CH_2_); 31.1 (CH_2_); 54.4 (CH_2_); 56.0 (CH_2_); 57.9 (CH_2_); 58.4 (CH_2_); 126.0 (CH_2_); 135.8 (C); 166.0 (C=O); IR (cm^−1^): 2954 ν(C-H); 1721 ν(C=O); 1640 ν(C=C); 1456 δ_as_(CH_2_); 1403 δ_s_(CH_3_); 1296 ν(C-O); 1157 ν(C-O); 1060 δ(CH_2_); 1011 δ(CH_2_); 936 δ(C-H); 808 δ(CH_2_); Elemental analysis calcd (%) for C_16_H_32_INO_2_: C, 48.37; H, 8.12; N, 3.53. Found (%): C, 48.43; H, 8.13; N, 3.50.

#### *N,N*-Diethyl-*N*-[2-(methacryloyloxy)ethyl]-*N*-octylammonium iodide (**[C_8_N_MA,22_]I**)

A mixture of 2-(diethylamino)ethyl methacrylate (DEAEMA, 27.789 g, 150.0 mmol), a slight excess of 1-iodooctane (39.621 g, 165.0 mmol) and phenothiazine (0.6 g, 3.0 mmol) as inhibitor in 15 mL acetonitrile was stirred under argon atmosphere at 75 °C for 3 days and under exclusion of UV irradiation. All volatile components were removed under reduced pressure. The precipitate was washed with ethyl ether and dried under vacuum. The product was received as a white powder (59.47 g, 93.2% yield). M.p.: 80.5 °C; ^1^H NMR (500 MHz, acetone-D6): δ (ppm) 0.88 (m, 3H, CH_3_); 1.32 (m, 6H, CH_2_); 1.46 (m, 10H, CH_3_, CH_2_); 1.91 (m, 2H, CH_2_); 1.96 (s, 3H, CH_3_); 3.64 (m, 2H, CH_2_); 3.76 (q, 4H, CH_2_); 4.06 (s, 2H, CH_2_); 4.73 (s, 2H, CH_2_); 5.74 (s, 1H, CH_2_); 6.15 (s, 1H, CH_2_); ^13^C NMR (125 MHz, acetone-D6): δ (ppm) 7.9 (CH_3_); 13.6 (CH_3_); 17.7 (CH_3_); 22.0 (CH_2_); 22.4 (CH_2_); 26.2 (CH_2_); 29.0 (CH_2_); 31.6 (CH_2_); 54.6 (CH_2_); 56.3 (CH_2_); 58.2 (CH_2_); 58.6 (CH_2_); 126.1 (CH_2_); 135.9 (C); 166.1 (C=O); IR (cm^−1^): 2928 ν(C-H); 1724 ν(C=O); 1640 ν(C=C); 1454 δ_as_(CH_2_); 1399 δ_s_(CH_3_); 1291 ν(C-O); 1154 ν(C-O); 1060 δ(CH_2_); 1009 δ(CH_2_); 936 δ(C-H); 806 δ(CH_2_); Elemental analysis calcd (%) for C_18_H_36_INO_2_: C, 50.82; H, 8.53; N, 3.29. Found (%): C, 50.51; H, 8.48; N, 3.27.

#### *N*-[2-(Acryloyloxy)ethyl]-*N,N,N*-triethylammonium iodide (**[C_2_N_A,22_]I**)

A mixture of 2-(diethylamino)ethyl acrylate (DEAEA, 25.686 g, 150.0 mmol), a slight excess of iodoethane (25.735 g, 165.0 mmol) and phenothiazine (0.6 g, 3.0 mmol) as inhibitor in 15 mL acetonitrile was stirred under argon atmosphere at 45 °C for 24 h and under exclusion of UV irradiation. All volatile components were removed under reduced pressure. The precipitate was washed with ethyl ether and dried under vacuum. The product was received as a white powder (48.33 g, 98.5% yield). M.p.: 93.1 °C; ^1^H NMR (500 MHz, acetone-D6): δ (ppm) 1.47 (t, 9H, CH_3_); 3.72 (q, 6H, CH_2_); 3.97 (m, 2H, CH_2_); 4.73 (s, 2H, CH_2_); 6.01 (dd, 1H, CH_2_); 6.24 (m, 1H, CH); 6.44 (dd, 1H, CH_2_); ^13^C NMR (125 MHz, acetone-D6): δ (ppm) 7.3 (CH_3_); 53.8 (CH_2_); 55.4 (CH_2_); 57.5 (CH_2_); 127.8 (CH); 131.6 (CH_2_); 164.8 (C=O); IR (cm^−1^): 2979 ν(C-H); 1719 ν(C=O); 1624 ν(C=C); 1461 δ_as_(CH_2_); 1401 δ_s_(CH_3_); 1271 ν(C-O); 1183 ν(C-O); 1086 δ(CH_2_); 1064 δ(CH_2_); 1020 δ(CH_2_); 998 δ(C-H); 960 δ(C-H); 808 δ(CH_2_); Elemental analysis calcd (%) for C_11_H_22_INO_2_: C, 40.38; H, 6.78; N, 4.28. Found (%): C, 40.61; H, 6.78; N, 4.52.

#### *N*-[2-(Acryloyloxy)ethyl]-*N*-butyl-*N,N*-diethylammonium iodide (**[C_4_N_A,22_]I**)

A mixture of 2-(diethylamino)ethyl acrylate (DEAEA, 15.945 g, 93.1 mmol), a slight excess of 1-iodobutane (18.849 g, 102.4 mmol) and phenothiazine (0.6 g, 3.0 mmol) as inhibitor in 15 mL acetonitrile was stirred under argon atmosphere at 75 °C for 2 days and under exclusion of UV irradiation. All volatile components were removed under reduced pressure. The precipitate was washed with ethyl ether and dried under vacuum. The product was received as a slightly yellowish powder (31.96 g, 96.6% yield). M.p.: 84.0 °C; ^1^H NMR (500 MHz, acetone-D6): δ (ppm) 1.02 (t, 3H, CH_3_); 1.47 (m, 8H, CH_3_, CH_2_); 1.89 (m, 2H, CH_2_); 3.63 (m, 2H, CH_2_); 3.75 (q, 4H, CH_2_); 4.01 (m, 2H, CH_2_); 4.74 (s, 2H, CH_2_); 6.02 (m, 1H, CH_2_); 6.24 (m, 1H, CH); 6.44 (m, 1H, CH_2_); ^13^C NMR (125 MHz, acetone-D6): δ (ppm) 7.6 (CH_3_); 13.1 (CH_2_); 19.5 (CH_2_); 23.8 (CH_2_); 54.5 (CH_2_); 56.1 (CH_2_); 57.7 (CH_2_); 58.3 (CH_2_); 127.8 (CH); 131.7 (CH_2_); 164.8 (C=O); IR (cm^−1^): 2965 ν(C-H); 1724 ν(C=O); 1622 ν(C=C); 1459 δ_as_(CH_2_); 1406 δ_s_(CH_3_); 1260 ν(C-O); 1188 ν(C-O); 1071 δ(CH_2_); 1022 δ(CH_2_); 965 δ(C-H); 806 δ(CH_2_); Elemental analysis calcd (%) for C_13_H_26_INO_2_: C, 43.95; H, 7.38; N, 3.94. Found (%): C, 44.18; H, 7.38; N, 4.00.

#### *N*-[2-(Acryloyloxy)ethyl]-*N,N*-diethyl-*N*-hexylammonium iodide (**[C_6_N_A,22_]I**)

A mixture of 2-(diethylamino)ethyl acrylate (DEAEA, 25.686 g, 150.0 mmol), a slight excess of 1-iodohexane (34.992 g, 165.0 mmol) and phenothiazine (0.6 g, 3.0 mmol) as inhibitor in 15 mL acetonitrile was stirred under argon atmosphere at 60 °C for 3 days and under exclusion of UV irradiation. All volatile components were removed under reduced pressure. The precipitate was washed with ethyl ether and dried under vacuum. The product was received as a white powder (55.26 g, 96.1% yield). M.p.: 81.1 °C; ^1^H NMR (500 MHz, acetone-D6): δ (ppm) 0.91 (t, 3H, CH_3_); 1.41 (m, 6H, CH_2_); 1.48 (t, 6H, CH_3_); 1.92 (m, 2H, CH_2_); 3.62 (q, 2H, CH_2_); 3.74 (s, 4H, CH_2_); 3.99 (m, 2H, CH_2_); 4.74 (s, 2H, CH_2_); 6.02 (m, 1H, CH_2_); 6.24 (m, 1H, CH); 6.45 (m, 1H, CH_2_); ^13^C NMR (125 MHz, acetone-D6): δ (ppm) 7.4 (CH_3_); 13.3 (CH_2_); 21.8 (CH_2_); 22.2 (CH_2_); 25.8 (CH_2_); 31.1 (CH_2_); 54.4 (CH_2_); 56.0 (CH_2_); 57.6 (CH_2_); 58.5 (CH_2_); 127.8 (CH); 131.6 (CH_2_); 164.8 (C=O); IR (cm^−1^): 2954 ν(C-H); 1721 ν(C=O); 1624 ν(C=C); 1456 δ(CH_2_); 1406 δ_s_(CH_3_); 1263 ν(C-O); 1188 ν(C-O); 1071 δ(CH_2_); 1022 δ(CH_2_); 971 δ(C-H); 936 δ(C-H); 806 δ(CH_2_); Elemental analysis calcd (%) for C_15_H_30_INO_2_: C, 47.00; H, 7.89; N, 3.65. Found (%): C, 47.05; H, 7.89; N, 3.66.

#### *N*-[2-(Acryloyloxy)ethyl]-*N,N*-diethyl-*N*-octylammonium iodide (**[C_8_N_A,22_]I**)

A mixture of 2-(diethylamino)ethyl acrylate (DEAEA, 25.686 g, 150.0 mmol), a slight excess of 1-iodooctane (39.621 g, 165.0 mmol) and phenothiazine (0.6 g, 3.0 mmol) as inhibitor in 15 mL acetonitrile was stirred under argon atmosphere at 60 °C for 3 days and under exclusion of UV irradiation. All volatile components were removed under reduced pressure. The precipitate was washed with ethyl ether and dried under vacuum. The product was received as a white powder (55.16 g, 89.4% yield). M.p.: 54.4 °C; ^1^H NMR (500 MHz, acetone-D6): δ (ppm) 0.89 (t, 3H, CH_3_); 1.33 (m, 6H, CH_2_); 1.47 (m, 10H, CH_3_, CH_2_); 1.92 (m, 2H, CH_2_); 3.62 (m, 2H, CH_2_); 3.75 (q, 4H, CH_2_); 4.01 (m, 2H, CH_2_); 4.74 (s, 2H, CH_2_); 6.01 (m, 1H, CH_2_); 6.24 (m, 1H, CH); 6.45 (m, 1H, CH_2_); ^13^C NMR (125 MHz, acetone-D6): δ (ppm) 7.5 (CH_3_); 13.5 (CH_2_); 21.9 (CH_2_); 22.4 (CH_2_); 26.2 (CH_2_); 28.9 (CH_2_); 31.6 (CH_2_); 54.5 (CH_2_); 56.1 (CH_2_); 57.7 (CH_2_); 58.5 (CH_2_); 127.8 (CH); 131.6 (CH_2_); 164.8 (C=O); IR (cm^−1^): 2926 ν(C-H); 1728 ν(C=O); 1640 ν(C=C); 1454 δ_as_(CH_2_); 1406 δ_s_(CH_3_); 1267 ν(C-O); 1188 ν(C-O); 1075 δ(CH_2_); 1009 δ(CH_2_); 978 δ(C-H); 804 δ(CH_2_); Elemental analysis calcd (%) for C_17_H_34_INO_2_: C, 49.64; H, 8.33; N, 4.40. Found (%): C, 49.46; H, 8.22; N, 3.35.

#### *N,N,N*-Triethyl-*N*-[2-(methacryloyloxy)ethyl]ammonium bis(trifluoromethanesulfonyl)imide (**[C_2_N_MA,22_]TFSI**)

The respective intermediate [C_2_N_MA,22_]I (22.180 g, 65.0 mmol) was dissolved in water (15 mL) and added to a solution of LiTFSI (19.501 g, 67.9 mmol) in water (15 mL). The ionic liquid precipitated instantly and the mixture was stirred further for 10 min at room temperature. The aqueous top layer was removed and the remaining organic layer was washed six times with water (40 mL), respectively. No iodide ions were detected in the last washing water by AgNO_3_ solution. The ionic liquid was diluted in acetone (40 mL) and dried over molecular sieves (10 g, 3 Å) for 24 h. The solution was filtrated (PTFE, 0.2 µm) and the acetone removed under reduced pressure. The ionic liquid was stirred at room temperature under vacuum (6 mbar) for 2 days to give the pure product as colorless oil (29.16 g, 90.7%yield). ^1^H NMR (500 MHz, CDCl3): δ (ppm) 1.28 (t, 9H, CH_3_); 1.87 (s, 3H, CH_3_); 3.32 (q, 6H, CH_2_); 3.53 (m, 2H, CH_2_); 4.45 (m, 2H, CH_2_); 5.62 (m, 1H, CH_2_); 6.03 (s, 1H, CH_2_); ^13^C NMR (125 MHz, CDCl3): δ (ppm) 7.3 (CH_3_); 18.0 (CH_2_); 54.0 (CH_2_); 55.3 (CH_2_); 57.3 (CH_2_); 120.1 (q, CF3); 127.3 (CH_2_); 135.1 (C); 166.4 (C=O); IR (cm^−1^): 2996 ν(C-H); 1726 ν(C=O); 1638 ν(C=C); 1457 δ_as_(CH_2_); 1399 δ_s_(CH_3_); 1349 ν(SO_2_); 1179 ν(C-O); 1135 ν(S=O); 1053 ν(S=O); 1002 δ(CH_2_); 742 δ_s_ (CF_3_); 654 δ(SNS); 614 δ_a_(SO_2_); Elemental analysis calcd (%) for C_14_H_24_F_6_N_2_O_6_S_2_: C, 34.01; H, 4.89; N, 5.67. Found (%): C, 34.11; H, 4.80; N, 5.73.

#### *One-pot synthesis of* **[C_2_N_MA,22_]TFSI**

DEAEMA (3.149 g, 17.0 mmol) and 1-iodoethane (2.917 g, 18.7 mmol) were added to a solution of LiTFSI (5.100 g, 17.8 mmol) in acetonitrile (5 mL). The mixture was stirred at elevated temperature (45 °C) for 16 h. All volatile components were removed under reduced pressure. The remaining oily phase was washed with water (120 mL in total) in six portions. The IL phase was dissolved in acetone (15 mL) and the solution was dried over molecular sieves (2.5 g, 3 Å) for 3 days. The solution was filtrated (PTFE, 0.2 µm) and the acetone was removed under reduced pressure. The ionic liquid was stirred at room temperature under vacuum (6 mbar) to give the pure product as colorless oil (5.56 g, 66.2% yield).

#### *N*-Butyl-*N,N*-diethyl-*N*-[2-(methacryloyloxy)ethyl]ammonium bis(trifluoromethanesulfonyl)imide (**[C_4_N_MA,22_]TFSI**)

The respective intermediate [C_4_N_MA,22_]I (24.003 g, 65.0 mmol) was suspended in water (30 mL) and the supernatant water was added to a solution of LiTFSI (19.501 g, 67.9 mmol) in water (15 mL). The undissolved intermediate salt was solved in acetone (15 mL) and added dropwise to the aqueous phase. The ionic liquid precipitated instantly and the mixture was stirred for 10 min at room temperature. Further processing was realized as described above for [C_2_N_MA,22_]TFSI to give the product as colorless oil (30.17 g, 88.8% yield). ^1^H NMR (500 MHz, CDCl3): δ (ppm) 0.94 (t, 3H, CH_3_); 1.30 (t, 6H, CH_3_); 1.36 (q, 2H, CH_2_); 1.62 (m, 2H, CH_2_); 1.90 (s, 3H, CH_3_); 3.18 (m, 2H, CH_2_); 3.35 (q, 4H, CH_2_); 3.57 (m, 2H, CH_2_); 4.47 (m, 2H, CH_2_); 5.64 (s, 1H, CH_2_); 6.06 (s, 1H, CH_2_); ^13^C NMR (125 MHz, CDCl3): δ (ppm) 7.4 (CH_3_); 13.3 (CH_3_); 18.0 (CH_3_); 19.4 (CH_2_); 23.5 (CH_2_); 54.5 (CH_2_); 55.8 (CH_2_); 57.2 (CH_2_); 58.4 (CH_2_); 119.8 (q, CF3); 127.2 (CH_2_); 135.0 (C); 166.4 (C=O); IR (cm^−1^): 2970 ν(C-H); 1724 ν(C=O); 1638 ν(C=C); 1459 δ_as_(CH_2_); 1349 ν(SO_2_); 1181 ν(C-O); 1135 ν(S=O); 1053 ν(S=O); 949 δ(C-H); 788 δ(CH_2_); 742 δ_s_ (CF_3_); 614 δ(SNS); Elemental analysis calcd (%) for C_16_H_28_F_6_N_2_O_6_S_2_: C, 36.78; H, 5.40; N, 5.36. Found (%): C, 37.10; H, 5.26; N, 5.37.

#### *N,N*-Diethyl-*N*-hexyl-*N*-[2-(methacryloyloxy)ethyl]ammonium bis(trifluoromethanesulfonyl)imide (**[C_6_N_MA,22_]TFSI**)

The respective intermediate [C_6_N_MA,22_]I (26.74 g, 65.0 mmol) was dissolved in acetone (40 mL) and added dropwise to a solution of LiTFSI (19.501 g, 67.9 mmol) in water (40 mL). The ionic liquid precipitated instantly and the mixture was stirred for 10 min at room temperature. Further processing was realized as described above for [C_2_N_MA,22_]TFSI to give the product as colorless oil (32.81 g, 91.7% yield). ^1^H NMR (500 MHz, CDCl3): δ (ppm) 0.85 (t, 3H, CH_3_); 1.30 (m, 12H, CH_3_, CH_2_); 1.63 (m, 2H, CH_2_); 1.90 (s, 3H, CH_3_); 3.17 (m, 2H, CH_2_); 3.36 (q, 4H, CH_2_); 3.57 (m, 2H, CH_2_); 4.47 (s, 2H, CH_2_); 5.64 (s, 1H, CH_2_); 6.06 (s, 1H, CH_2_); ^13^C NMR (125 MHz, CDCl3): δ (ppm) 7.3 (CH_3_); 13.7 (CH_3_); 18.0 (CH_3_); 21.6 (CH_2_); 22.3 (CH_2_); 25.7 (CH_2_); 30.9 (CH_2_); 54.4 (CH_2_); 55.8 (CH_2_); 57.2 (CH_2_); 58.6 (CH_2_); 119.8 (q, CF3); 127.2 (CH_2_); 135.0 (C); 166.3 (C=O); IR (cm^−1^): 2963 ν(C-H); 2932 ν(C-H); 2864 ν(C-H); 1724 ν(C=O); 1638 ν(C=C); 1459 δ_as_(CH_2_); 1399 δ_s_(CH_3_); 1348 ν(SO_2_); 1181 ν(C-O); 1135 ν(S=O); 1055 ν(S=O); 949 δ(C-H); 788 δ(CH_2_); 742 δ_s_ (CF_3_); 654 δ(SNS); 616 δ_a_(SO_2_); Elemental analysis calcd (%) for C_18_H_32_F_6_N_2_O_6_S_2_: C, 39.27; H, 5.86; N, 5.09. Found (%): C, 39.48; H, 5.74; N, 5.07.

#### *N,N*-Diethyl-*N*-[2-(methacryloyloxy)ethyl]-*N*-octylammonium bis(trifluoromethanesulfonyl)imide (**[C_8_N_MA,22_]TFSI**)

The IL was prepared accordingly to the procedure described above for [C_6_N_MA,22_]TFSI starting from [C_8_N_MA,22_]I (27.65 g, 65.0 mmol) in acetone (40 mL) and LiTFSI (19.501 g, 67.9 mmol) in water (40 mL). The product was received as colorless oil (34.11 g, 90.7% yield). ^1^H NMR (500 MHz, CDCl3): δ (ppm) 0.87 (t, 3H, CH_3_); 1.27 (m, 6H, CH_3_); 1.33 (m, 10H, CH_2_); 1.65 (s, 2H, CH_2_); 1.93 (s, 3H, CH_3_); 3.20 (m, 2H, CH_2_); 3.38 (q, 4H, CH_2_); 3.59 (m, 2H, CH_2_); 4.50 (m, 2H, CH_2_); 5.67 (s, 1H, CH_2_); 6.09 (s, 1H, CH_2_); ^13^C NMR (125 MHz, CDCl3): δ (ppm) 7.4 (CH_3_); 14.0 (CH_3_); 18.0 (CH_3_); 21.7 (CH_2_); 22.5 (CH_2_); 26.1 (CH_2_); 28.9 (CH_2_); 31.5 (CH_2_); 54.5 (CH_2_); 55.8 (CH_2_); 57.2 (CH_2_); 58.6 (CH_2_); 119.8 (q, CF3); 127.2 (CH_2_); 135.0 (C); 166.4 (C=O); IR (cm^−1^): 2959 ν(C-H); 2932 ν(C-H); 2857 ν(C-H); 1726 ν(C=O); 1638 ν(C=C); 1461 δ_as_(CH_2_); 1399 δ_s_(CH_3_); 1351 ν(SO_2_); 1183 ν(C-O); 1135 ν(S=O); 1055 ν(S=O); 947 δ(C-H); 788 δ(CH_2_); 742 δ_s_ (CF_3_); 654 δ(SNS); 616 δ_a_(SO_2_); Elemental analysis calcd (%) for C_20_H_36_F_6_N_2_O_6_S_2_: C, 41.52; H, 6.27; N, 4.84. Found (%): C, 41.64; H, 6.05; N, 4.78.

#### *N*-[2-(Acryloyloxy)ethyl]-*N,N,N*-triethylammonium bis(trifluoromethanesulfonyl)imide (**[C_2_N_A,22_]TFSI**)

The IL was prepared accordingly to the procedure described above for [C_2_N_MA,22_]TFSI starting from [C_2_N_A,22_]I (24.451 g, 75.0 mmol) in water (15 mL) and LiTFSI (22.501 g, 68.38 mmol) in water (15 mL). The product was received as colorless oil (30.64 g, 85.0% yield). ^1^H NMR (500 MHz, acetone-D6): δ (ppm) 1.44 (t, 9H, CH_3_); 3.62 (q, 6H, CH_2_); 3.83 (m, 2H, CH_2_); 4.67 (s, 2H, CH_2_); 6.00 (dd, 1H, CH_2_); 6.22 (m, 1H, CH); 6.42 (dd, 1H, CH_2_); ^13^C NMR (125 MHz, acetone-D6): δ (ppm) 6.9 (CH_3_); 53.7 (CH_2_); 55.1 (CH_2_); 57.3 (CH_2_); 120.1 (q, CF3); 127.6 (CH); 131.7 (CH_2_); 164.8 (C=O); IR (cm^−1^): 2996 ν(C-H); 1730 ν(C=O); 1638 ν(C=C); 1461 δ_as_(CH_2_); 1410 δ_s_(CH_3_); 1349 ν(SO_2_); 1177 ν(C-O); 1135 ν(S=O); 1053 ν(S=O); 996 δ(CH_2_); 788 δ(CH_2_); 742 δ_s_ (CF_3_); 654 δ(SNS); 614 δ_a_(SO_2_); Elemental analysis calcd (%) for C_13_H_22_F_6_N_2_O_6_S_2_: C, 32.50; H, 4.62; N, 5.83. Found (%): C, 32.45; H, 4.40; N, 5.83.

#### *N*-[2-(Acryloyloxy)ethyl]-*N*-butyl-*N,N*-diethylammonium bis(trifluoromethanesulfonyl)imide (**[C_4_N_A,22_]TFSI**)

The IL was prepared accordingly to the procedure described above for [C_2_N_MA,22_]TFSI starting from [C_4_N_A,22_]I (26.645 g, 75.0 mmol) in water (20 mL) and LiTFSI (22.501 g, 68.38 mmol) in water (15 mL). The product was received as colorless oil (30.49 g, 80.5% yield). ^1^H NMR (500 MHz, acetone-D6): δ (ppm) 1.00 (s, 3H, CH_3_); 1.43 (s, 8H, CH_3_, CH_2_); 1.83 (s, 2H, CH_2_); 3.48 (s, 2H, CH_2_); 3.62 (s, 4H, CH_2_); 3.84 (s, 2H, CH_2_); 4.67 (s, 2H, CH_2_); 6.00 (m, 1H, CH_2_); 6.21 (m, 1H, CH); 6.42 (m, 1H, CH_2_); ^13^C NMR (125 MHz, acetone-D6): δ (ppm) 7.0 (CH_3_); 12.9 (CH_2_); 19.4 (CH_2_); 23.4 (CH_2_); 54.2 (CH_2_); 55.7 (CH_2_); 57.3 (CH_2_); 58.1 (CH_2_); 120.1 (q, CF3); 127.6 (CH); 131.7 (CH_2_); 164.8 (C=O); IR (cm^−1^): 2961 ν(C-H); 2935 ν(C-H); 2866 ν(C-H); 1730 ν(C=O); 1638 ν(C=C); 1463 δ_as_(CH_2_); 1408 δ_s_(CH_3_); 1351 ν(SO_2_); 1179 ν(C-O); 1135 ν(S=O); 1055 ν(S=O); 790 δ(CH_2_); 742 δ_s_ (CF_3_); 654 δ(SNS); 616 δ_a_(SO_2_); Elemental analysis calcd (%) for C_15_H_26_F_6_N_2_O_6_S_2_: C, 35.43; H, 5.15; N, 5.51. Found (%): C, 35.45; H, 5.15; N, 5.57.

#### *N*-[2-(Acryloyloxy)ethyl]-*N,N*-diethyl-*N*-hexylammonium bis(trifluoromethanesulfonyl)imide (**[C_6_N_A,22_]TFSI**)

The IL was prepared accordingly to the procedure described above for [C_6_N_MA,22_]TFSI starting from [C_6_N_A,22_]I (24.915 g, 65.0 mmol) in acetone (40 mL) and LiTFSI (19.501 g, 67.9 mmol) in water (30 mL). The product was received as colorless oil (31.20 g, 89.5% yield). ^1^H NMR (500 MHz, CDCl3): δ (ppm) 0.89 (s, 3H, CH_3_); 1.33 (s, 12H, CH_3_, CH_2_); 1.66 (s, 2H, CH_2_); 3.21 (m, 2H, CH_2_); 3.38 (m, 4H, CH_2_); 3.60 (s, 2H, CH_2_); 4.52 (s, 2H, CH_2_); 5.96 (m, 1H, CH_2_); 6.11 (m, 1H, CH); 6.44 (m, 1H, CH_2_); ^13^C NMR (125 MHz, CDCl3): δ (ppm) 7.4 (CH_3_); 13.7 (CH_2_); 21.7 (CH_2_); 22.3 (CH_2_); 25.8 (CH_2_); 31.0 (CH_2_); 54.5 (CH_2_); 55.8 (CH_2_); 56.9 (CH_2_); 58.7 (CH_2_); 119.8 (q, CF3); 126.9 (CH); 132.8 (CH_2_); 165.1 (C=O); IR (cm^−1^): 2972 ν(C-H); 2883 ν(C-H); 1732 ν(C=O); 1637 ν(C=C); 1463 δ_as_(CH_2_); 1408 δ_s_(CH_3_); 1349 ν(SO_2_); 1179 ν(C-O); 1135 ν(S=O); 1053 ν(S=O); 790 δ(CH_2_); 740 δ_s_ (CF_3_); 653 δ(SNS); 614 δ_a_(SO_2_); Elemental analysis calcd (%) for C17H30F6N2O6S2: C, 38.06; H, 5.64; N, 5.22. Found (%): C, 37.70; H, 5.63; N, 5.13.

#### *N*-[2-(Acryloyloxy)ethyl]-*N,N*-diethyl-*N*-octylammonium bis(trifluoromethanesulfonyl)imide (**[C_8_N_A,22_]TFSI**)

The IL was prepared accordingly to the procedure described above for [C_6_N_MA,22_]TFSI starting from [C_8_N_A,22_]I (26.739 g, 65.0 mmol) in acetone (40 mL) and LiTFSI (19.501 g, 67.9 mmol) in water (30 mL). The product was received as colorless oil (32.77 g, 89.3% yield). ^1^H NMR (500 MHz, CDCl3): δ (ppm) 0.88 (t, 3H, CH_3_); 1.28 (m, 6H, CH_2_); 1.34 (t, 10H, CH_3_, CH_2_); 1.66 (s, 2H, CH_2_); 3.20 (m, 2H, CH_2_); 3.39 (q, 4H, CH_2_); 3.60 (m, 2H, CH_2_); 4.52 (m, 2H, CH_2_); 5.96 (dd, 1H, CH_2_); 6.12 (m, 1H, CH); 6.45 (dd, 1H, CH_2_); ^13^C NMR (125 MHz, CDCl3): δ (ppm) 7.4 (CH_3_); 14.0 (CH_2_); 21.8 (CH_2_); 22.5 (CH_2_); 26.1 (CH_2_); 28.9 (CH_2_); 31.5 (CH_2_); 54.5 (CH_2_); 55.8 (CH_2_); 56.9 (CH_2_); 58.7 (CH_2_); 119.8 (q, CF3); 126.9 (CH); 132.9 (CH_2_); 165.1 (C=O); IR (cm^−1^): 2959 ν(C-H); 2932 ν(C-H); 2860 ν(C-H); 1732 ν(C=O); 1638 ν(C=C); 1465 δ_as_(CH_2_); 1408 δ_s_(CH_3_); 1349 ν(SO_2_); 1179 ν(C-O); 1135 ν(S=O); 1055 ν(S=O); 790 δ(CH_2_); 742 δ_s_ (CF_3_); 656 δ(SNS); 616 δ_a_(SO_2_); Elemental analysis calcd (%) for C_19_H_34_F_6_N_2_O_6_S_2_: C, 40.42; H, 6.07; N, 4.96. Found (%): C, 41.04; H, 6.01; N, 5.06.

## Figures and Tables

**Table 1:** Selected reaction conditions and yields of the intermediate products.

Product	Solvent	Reaction Time	Temperature	Alkyl Halide Excess	Yield
[C_2_N_MA,22_]Br	Acetone	6 d	47.0 °C	1.4	60.7%
[C_4_N_MA,22_]Br	Acetone	6 d	47.0 °C	1.4	7.0%
[C_6_N_MA,22_]Br	Acetone	11 d	56.0 °C	1.4	51.5% ^a^
[C_4_N_MA,22_]Br	CH_2_Cl_2_	16 h	39.6 °C	1.2	0.0%
[C_6_N_MA,22_]I	Acetone	5 d	56.0 °C	1.4	66.3%
[C_6_N_MA,22_]Br	Acetonitrile	6 d	56.0 °C	1.4	54.0%
[C_2_N_MA,22_]I	Acetonitrile	1 d	45.0 °C	1.1	96.4%
[C_4_N_MA,22_]I	Acetonitrile	3 d	60.0 °C	1.1	97.5%
[C_6_N_MA,22_]I	Acetonitrile	3 d	60.0 °C	1.1	95.9%

^a 1^H NMR spectroscopy revealed that approx. 25% of the double bonds were degraded during the reaction.

**Table 2:** Particular yields of all reaction steps and resulting overall yields for the ILs.

Precursor	Yield [%]	Ionic Liquid	Yield [%]	Overall Yield [%]
[C_2_N_MA,22_]I	96.4	[C_2_N_MA,22_]TFSI	90.7	87.4
[C_4_N_MA,22_]I	97.5	[C_4_N_MA,22_]TFSI	88.8	86.6
[C_6_N_MA,22_]I	95.9	[C_6_N_MA,22_]TFSI	91.7	87.9
[C_8_N_MA,22_]I	93.2	[C_8_N_MA,22_]TFSI	90.7	84.5
[C_2_N_A,22_]I	98.5	[C_2_N_A,22_]TFSI	85.0	83.7
[C_4_N_A,22_]I	96.6	[C_4_N_A,22_]TFSI	80.5	77.8
[C_6_N_A,22_]I	96.1	[C_6_N_A,22_]TFSI	89.5	86.0
[C_8_N_A,22_]I	89.4	[C_8_N_A,22_]TFSI	89.3	79.8

**Table 3:** Parameters from linear fit of density values (*y* = *m* × *x* + *n*).

IONIC Liquid	*m*	*n*/g∙cm^−3^
[C_2_N_MA,22_]TFSI	−8.86882 × 10^−4^	1.40202
[C_4_N_MA,22_]TFSI	−8.70826 × 10^−4^	1.34746
[C_6_N_MA,22_]TFSI	−8.44608 × 10^−4^	1.30603
[C_8_N_MA,22_]TFSI	−8.29652 × 10^−4^	1.27181
[C_2_N_A,22_]TFSI	−8.76545 × 10^−4^	1.42503
[C_4_N_A,22_]TFSI	−8.64216 × 10^−4^	1.36936
[C_6_N_A,22_]TFSI	−8.40463 × 10^−4^	1.32206
[C_8_N_A,22_]TFSI	−8.18980 × 10^−4^	1.28568

**Table 4:** Melting points (*T*_m_) and glass transition temperatures (*T*_g_) of the products. Standard uncertainties *u* for values from this work are *u*(*T*_m_) = 2 K; *u*(*T*_g_) = 2 K.

Halogen Intermediate Salt			Ionic Liquid				
Entry	Product	Onset [K]	Entry	Product	T_g,1_ [K] ^1^	T_g,2_ [K] ^2^	Standard Deviation ^2^ s
1	[C_2_N_MA,22_]I	339.9	12	[C_2_N_MA,22_]TFSI	219.2	223.0	0.3
2	[C_4_N_MA,22_]I	353.3	13	[C_4_N_MA,22_]TFSI	216.8	223.2	1.2
3	[C_6_N_MA,22_]I	332.9	14	[C_6_N_MA,22_]TFSI	215.2	220.4	1.0
4	[C_8_N_MA,22_]I	317.7	15	[C_8_N_MA,22_]TFSI	213.2	218.4	0.2
5	[C_2_N_A,22_]I	330.3	16	[C_2_N_A,22_]TFSI	211.7	215.5	0.9
6	[C_4_N_A,22_]I	321.2	17	[C_4_N_A,22_]TFSI	214.5	218.2	1.1
7	[C_6_N_A,22_]I	318.3	18	[C_6_N_A,22_]TFSI	211.6	216.1	0.4
8	[C_8_N_A,22_]I	291.6	19	[C_8_N_A,22_]TFSI	212.6	214.4	0.6
9	[C_2_N_MA,22_]Br	348.4					
10	[C_4_N_MA,22_]Br	345.1					
11	[C_6_N_MA,22_]Br	339.9					

^1^ Measurement two weeks after synthesis and storage at −40 °C diluted in acetone (50 wt%). Acetone was completely removed under vacuum before measurement. ^2^ Measurements after eight weeks of storage at same conditions as before.

**Table 5:** Experimentally measured values of ionic conductivity (*κ*), density (*d*), viscosity (*η*) and calculated molar conductivity (*Λ*_m_) at 25 °C and at pressure *p* = 0.1 MPa. Standard uncertainties *u* for values from this work are *u*(*d*) = 0.001 g cm^−3^; *u*(*η*) = 0.05·*η*; *u*(*κ*) = 0.03·*κ*; *u*(*p*) = 5 kPa; *u* (*Λ*_m_) = 0.06∙*Λ*_m_; *u*(*T_κ_*) = 0.1 K; *u*(*T_η_*) = 0.1 K; *u*(*T_d_*) = 0.01 K (specified temperature during conductivity (*T_κ_*), density (*T_d_*) and viscosity (*T_η_*) measurement).

Ionic Liquid	Ionic Conductivity [mS cm^−1^]	Molar Conductivity [mS cm^2^ mol^−1^]	Density [g cm^−3^]	Viscosity [Pa s]
[C_2_N_MA,22_]TFSI	0.211	75.7	2.790	0.905
[C_4_N_MA,22_]TFSI	0.112	44.2	2.537	1.395
[C_6_N_MA,22_]TFSI	0.093	39.8	2.334	1.522
[C_8_N_MA,22_]TFSI	0.080	36.8	2.162	1.425
[C_2_N_A,22_]TFSI	0.264	90.3	2.920	0.762
[C_4_N_A,22_]TFSI	0.136	51.1	2.650	1.477
[C_6_N_A,22_]TFSI	0.122	50.3	2.425	1.284
[C_8_N_A,22_]TFSI	0.103	46.1	2.241	1.246

**Table 6:** Comparison of experimentally determined *T*_g_ and calculated *T*_0_ from VFT fit. Standard uncertainties *u* for values from this work are *u*(*T*_g_) = 2 K.

Ionic Liquid	*T*_g_ [K] ^1,2^	*T*_0_ [K] ^3^	*T*_g_/*T*_0_
[C_2_N_MA,22_]TFSI	219.2	201.8	1.09
[C_4_N_MA,22_]TFSI	216.8	208.7	1.04
[C_6_N_MA,22_]TFSI	215.2	197.7	1.09
[C_8_N_MA,22_]TFSI	213.2	200.0	1.07
[C_2_N_A,22_]TFSI	211.7	186.8	1.13
[C_4_N_A,22_]TFSI	214.5	202.7	1.06
[C_6_N_A,22_]TFSI	211.6	193.7	1.09
[C_8_N_A,22_]TFSI	212.6	190.6	1.11

^1^ Experimentally measured values are provided. ^2^ The measurement was performed two weeks after synthesis and storage at −40 °C diluted in acetone (50 wt%). Acetone was completely removed under vacuum before measurement. ^3^ Values are calculated from VFT fits of ionic conductivity values (Figure 7b).

**Table 7:** Calculated Flow activation energies from Arrhenius fit are shown (20 °C–60 °C).

Ionic Liquid	*E*_η_ [kJ mol^−1^]
[C_2_N_MA,22_]TFSI	54.9 ± 1.5
[C_4_N_MA,22_]TFSI	59.7 ± 1.3
[C_6_N_MA,22_]TFSI	58.1 ± 1.1
[C_8_N_MA,22_]TFSI	56.7 ± 1.0
[C_2_N_A,22_]TFSI	50.3 ± 1.0
[C_4_N_A,22_]TFSI	57.1 ± 1.1
[C_6_N_A,22_]TFSI	54.0 ± 0.9
[C_8_N_A,22_]TFSI	53.1 ± 0.8
